# Effects of muscle cooling on kinetics of pulmonary oxygen uptake and muscle deoxygenation at the onset of exercise

**DOI:** 10.14814/phy2.13910

**Published:** 2018-10-31

**Authors:** Hitoshi Wakabayashi, Mizuki Osawa, Shunsaku Koga, Ke Li, Hiroyuki Sakaue, Yasuo Sengoku, Hideki Takagi

**Affiliations:** ^1^ Faculty of Engineering Hokkaido University Sapporo Japan; ^2^ Faculty of Health and Sport Sciences University of Tsukuba Tsukuba Japan; ^3^ Applied Physiology Laboratory Kobe Design University Kobe Japan

**Keywords:** Cold skeletal muscle, glycolytic metabolism, near‐infrared spectroscopy

## Abstract

This study investigated effects of skeletal muscle cooling on the metabolic response and kinetics of pulmonary oxygen uptake ($\dot{V}$O_2_) and skeletal muscle deoxygenation during submaximal exercise. In the cooling condition (C), after immersion of the lower body into 12°C water for 30 min, eight healthy males performed 30‐min cycling exercise at the lactate threshold while undergoing thigh cooling by a water‐circulating pad. In the normal condition (N) as control, they conducted the same exercise protocol without cooling. Blood lactate concentration was significantly higher in C than N at 10 min after onset of exercise (4.0 ± 1.7 and 2.4 ± 1.2 mmol/L in C and N, *P* < 0.05). The percent change in the tissue oxygen saturation of the vastus lateralis, measured by a near‐infrared spectroscopy, was significantly lower in C at 2, 8, 10, and 20 min after the exercise onset compared with N (*P* < 0.05). The percent change in deoxy hemoglobin+myoglobin concentration (Deoxy[Hb+Mb]) showed a transient peak at the onset of exercise and significantly higher value in C at 10, 20, and 30 min after the exercise onset (*P* < 0.05). Compared to N, slower $\dot{V}$O_2_ kinetics (mean response time) was observed in C (45.6 ± 7.8 and 36.1 ± 7.7 sec in C and N, *P* < 0.05). The mean response time in C relative to N was significantly correlated with the transient peak of Deoxy[Hb+Mb] in C (*r* = 0.84, *P* < 0.05). These results suggest that lower oxygen delivery to the hypothermic skeletal muscle might induce greater glycolytic metabolism during exercise and slower $\dot{V}$O_2_ kinetics at the onset of exercise.

## Introduction

There are many situations where humans perform in cold environments during sports events, winter outdoor occupations, and natural disasters like flooding and tsunamis. Cold‐induced impairment of exercise performance including lower maximal force, velocity, and muscle endurance with lower skeletal muscle temperature (*T*_muscle_) has been reported and summarized in several articles (Clarke et al. 1958; Heus et al. 1995; Racinais and Oksa 2010; Wakabayashi et al. 2015). The physiological mechanism for the performance impairment induced by cold has been studied extensively in relation to neuromuscular function using electromyography (EMG). The lower shift of EMG frequency with lower *T*_muscle_ is generally observed during exercise in cold (Bigland‐Ritchie et al. 1981; Mucke and Heuer 1989; Oksa et al. 1997; Petrofsky and Laymon 2005; Wakabayashi et al. 2017) and is associated with a reduction of the muscle conduction velocity (Bigland‐Ritchie et al. 1981). Because of the lower contractility of each muscle fiber with low *T*_muscle_, greater numbers of motor units are recruited to maintain the same work load in thermoneutral condition (Mallette et al. 2018).

Some studies have undertaken the measurement of pulmonary oxygen uptake ($\dot{V}$O_2_) to evaluate the effect of low *T*_muscle_ on the whole body metabolism. Shiojiri et al. (1997) suggested that the slower pulmonary $\dot{V}$O_2_ response with low *T*_muscle_ at the onset of exercise was due to decreased O_2_ extraction and/or impairment of oxidative reactions in the cold skeletal muscle. However, due to the lack of direct measurement of muscle metabolism, it is still not clear how hypothermic skeletal muscle contributes to delayed $\dot{V}$O_2_ kinetics. Bowen et al. (2013) investigated the association between delayed $\dot{V}$O_2_ kinetics in hypoxic conditions and the transient peak of the skeletal muscle deoxygenation measured by near‐infrared spectroscopy (NIRS). Similar to what is present with hypoxia (Bowen et al. 2013), in cold muscle, transient deoxygenation reflecting lower O_2_ delivery relative to the muscle O_2_ consumption would be expected because of the lower muscle perfusion with blood (i.e., lower O_2_ delivery) in the cold (Rennie et al. 1980; Thorsson et al. 1985; Gregson et al. 2011). Additionally, fast‐twitch fiber recruitment and anaerobic metabolism are expected to be increased in the cold (Rome et al. 1992; Fujimoto et al. 2016). It is known that larger proportion of fast‐twitch fiber slows $\dot{V}$O_2_ kinetics in vitro (Crow and Kushmerick 1982) and in vivo (Barstow et al. 1996; Pringle et al. 2003). Thus, fast‐twitch fiber recruitment with hypothermic skeletal muscle could also delay $\dot{V}$O_2_ kinetics, although these possibilities have yet to be examined.

Accordingly, the purpose of this study was to investigate the effect of lower *T*_muscle_ on the muscle and whole body metabolic response during lactate threshold (LT) exercise in human on cold exposure. The LT work rate was chosen for detecting difference between cold and normal conditions, even with a slight recruitment of glycolytic metabolism in cold. It was hypothesized that, in comparison with normothermic condition, greater blood lactate concentration would be observed with hypothermic skeletal muscle, and transient peak of the skeletal muscle deoxygenation would result in slower $\dot{V}$O_2_ kinetics at the onset of exercise in cold.

## Methods

### Participants

Eight healthy males (mean ± standard deviation height: 173.4 ± 5.4 cm, body weight: 71.7 ± 12.4 kg) participated in this study. They were informed of the experimental procedures and gave their written consent before participation. All experimental procedures in this study were designed according to the principle of the Helsinki Declaration and approved by the Human Subjects Committee of the University of Tsukuba.

### Procedures

Prior to the main experiment, participants performed an incremental exercise test for assessing their LT using a bicycle ergometer (PowerMax VII, Konami). Participants performed an intermittent incremental cycling protocol consisting of 4‐min pedaling at 60 rpm and 2‐min rest interval. They started the first stage from 1.0 kp and gradually increased intensity by 0.3 kp every stage until their blood lactate concentration (La) was higher than 4.0 mmol/L. During the stage interval, capillary blood was sampled from their fingertip. Based on the La of each stage, the LT work rate was determined by the linear regression fitting model with least mean squared error using Lactate‐E software (Newell et al. 2007).

The participants performed 30‐min constant work rate exercise in both cooling (C) and normal (N) condition in random order on separate days. They sat on the seat of the ergometer (PowerMax VII, Konami) for 3 min for measuring resting baseline values. In C, participants underwent pre‐cooling by immersing their lower body into 12°C water for 30 min while remaining at rest, and then exited the immersion pool and performed 30‐min cycling exercise at the LT work rate at 60 rpm with cooling applied to their thighs. Both thighs were wrapped by several water perfusion cooling pads, through which 5°C water was circulated using a thermostatic bath system (LTB‐400, ASONE). In N, they conducted a similar exercise protocol without any cooling. The LT work rate was chosen for clearly detecting the recruitment of anaerobic metabolism in C compared to N.

### Measurements and analysis

The vastus lateralis *T*_muscle_ was estimated continuously, except during immersion, using a deep body temperature monitor (CM‐210, TERUMO) which detects the tissue temperature 5–10 mm below the skin surface using the zero‐heat‐flow‐method (Yamakage and Namiki 2003). This monitor measures skin surface temperature beneath a thermal insulating pad containing a heater, which equilibrates the skin temperature with the deep tissue temperature when heat flow from the skin is maintained at zero. The consistency between *T*_muscle_ measured using a needle thermocouple and the zero‐heat‐flow‐method was evaluated (Togawa et al. 1976). The skin temperature on the thigh (*T*_thigh_) was also measured using a thermistor sensor (PD‐K161, TERUMO).

Changes in the concentrations of oxygenated and deoxygenated hemoglobin and myoglobin (Oxy[Hb+Mb] and Deoxy[Hb+Mb]) in the vastus lateralis muscle were continuously measured using spatially resolved near‐infrared spectroscopy (NIRS) (BOM‐L1TR, Omegawave). The data were sampled using an A/D converter (Powerlab/16SP, AD Instruments) and recorded at 0.1 second intervals using a laptop computer. The optodes of the NIRS were housed in an optically dense black vinyl holder to ensure distance between an illuminant and two detectors at 20 (detector 1) and 40 mm (detector 2), respectively. The illuminant generated three wavelengths of near‐infrared light (780, 810 and 830 nm). The optodes were attached to the skin above the vastus lateralis using double‐sided adhesive tape. The Oxy[Hb+Mb] and Deoxy[Hb+Mb] concentrations only in the deep tissue region (Oxy[Hb+Mb]_2‐1_ and Deoxy[Hb+Mb]_2‐1_) were calculated from the [Hb+Mb] concentrations measured at the two detectors (detector 1 and 2) using the following formulae (Kashima 2003).

$$\begin{array}{cc} \text{Oxy}{\left[\text{Hb}+\text{Mb}\right]}_{2-1} & =(\text{Oxy}{\left[\text{Hb}+\text{Mb}\right]}_{2}\times L_{2}\\ & \;-\text{Oxy}{\left[\text{Hb}+\text{Mb}\right]}_{1}\times L_{1})/\left(L_{2}-L_{1}\right) \end{array}$$

$$\begin{array}{cc} \text{Deoxy}{\left[\text{Hb}+\text{Mb}\right]}_{2-1} & =(\text{Deoxy}{\left[\text{Hb}+\text{Mb}\right]}_{2}\times L_{2}\\ & \;-\text{Deoxy}{\left[\text{Hb}+\text{Mb}\right]}_{1}\times L_{1})/\\ & \;(L_{2}-L_{1}) \end{array}$$

where *L*_1_ and *L*_2_ represent length from the illuminant to the detector 1 and 2, respectively. This method enabled us to focus on the muscle oxygenation excluding the effect of the skin blood flow on [Hb+Mb] concentrations (Ando et al. 2010). Total [Hb+Mb] and tissue O_2_ saturation (StO_2_) was calculated by the following formulas.

Total[Hb+Mb]=Oxy[Hb+Mb]+Deoxy[Hb+Mb]

$${\text{StO}}_{2}=\text{Oxy}\left[\text{Hb}+\text{Mb}\right]/\text{Total}\left[\text{Hb}+\text{Mb}\right]\times 100$$

Percent change of Oxy[Hb+Mb], Deoxy[Hb+Mb], total[Hb+Mb] and StO_2_ from the baseline period before cooling was calculated for the following analyses, since the NIRS technique cannot measure the absolute [Hb+Mb] concentration and reference values for both conditions are set at thermoneutral baseline.

La concentration was measured at baseline, every 2 min in the first 10‐min exercise and every 10 min in the latter 20 min using a portable lactate analyzer (Lactate Pro LT‐1710, Arkray Inc). Breath‐by‐breath $\dot{V}$O_2_ was continuously measured by an automated respirometer (Metalyzer 3B, Cortex) and was interpolated at 5‐sec intervals. The kinetics of $\dot{V}$O_2_ at the onset of exercise was analyzed using the first 6‐min data excluding the initial 20 sec, since this study focused on the primary phase reflecting the kinetics of O_2_ consumption in the exercising muscle. To reduce the breath‐by‐breath $\dot{V}$O_2_ fluctuation, the data were smoothed using a 3‐point moving average. The $\dot{V}$O_2_ response was fitted to the following exponential equation using nonlinear least squares regression. The fitting of the exponential curve and 3‐point moving average for each participant was evaluated by coefficient of determination (*R* ^2^).

$$\dot{V}O_{2}\left(t\right)=\dot{V}O_{2(\text{base})}+\text{Amp}\left(1-{\text{exp}}^{-(t-\mathrm{TD})/\tau }\right)$$

where $\dot{V}$O_2 (base)_: $\dot{V}$O_2_ at the baseline before exercise onset, Amp: amplitude between the baseline and steady state (6 min after onset of exercise), TD: time delay, *τ*: time constant of the exponential function. Mean response time (MRT) was calculated according to the following formulae (Sietsema et al. 1994).

$$\text{MRT}=O_{2}\;\text{deficit}/\left(\dot{V}O_{2(6)}-\dot{V}O_{2(\text{base})}\right)$$

where O_2_ deficit: subtracting 6‐min cumulative $\dot{V}$O_2_ from the required $\dot{V}$O_2_, i.e., the product of $\dot{V}$O_2_ at 6 min and the duration (6 min), $\dot{V}$O_2 (6)_: $\dot{V}$O_2_ at 6 min after exercise onset. The increment in $\dot{V}$O_2_ between minutes 2 and 6 (∆$\dot{V}$O_2 (6–2)_) was calculated for assessing the slow component of $\dot{V}$O_2_ kinetics (Whipp and Wasserman 1986). Additionally, $\dot{V}$O_2_ amplitude between baseline and 30 min after the onset of exercise (∆$\dot{V}$O_2 (30–base)_) was calculated.

### Statistical analysis

Data from time‐course measurements (*T*_muscle_, *T*_thigh_, $\dot{V}$O_2_, La, percent change of Oxy[Hb+Mb], Deoxy[Hb+Mb], total[Hb+Mb] and StO_2_) were evaluated using repeated measures two‐way (condition × time) analysis of variance (ANOVA). If Mauchly's sphericity test was not satisfied, the degrees of freedom were adjusted by Greenhouse‐Geisser's *ε*. Paired Student's t‐test with Holm's multiple comparisons adjustment (Holm 1979) was conducted between conditions at each time point. Parameters of $\dot{V}$O_2_ kinetics (baseline, Amp, TD, *τ*, MRT, O_2_ deficit and ∆$\dot{V}$O_2 (6–2)_ and ∆$\dot{V}$O_2 (30–base)_) were compared between conditions using paired *t*‐test. Pearson correlation coefficient was calculated to examine the relationships between parameters of $\dot{V}$O_2_ kinetics and muscle deoxygenation. Cohen's *d* and partial *η* ^2^ (*η*_*p*_^2^) was calculated for assessing effect size for paired t‐test and ANOVA, respectively. Statistical significance was established at *P* < 0.05. All data are presented as mean values and standard deviation (SD).

## Results

### Body temperature

**Figure 1:**
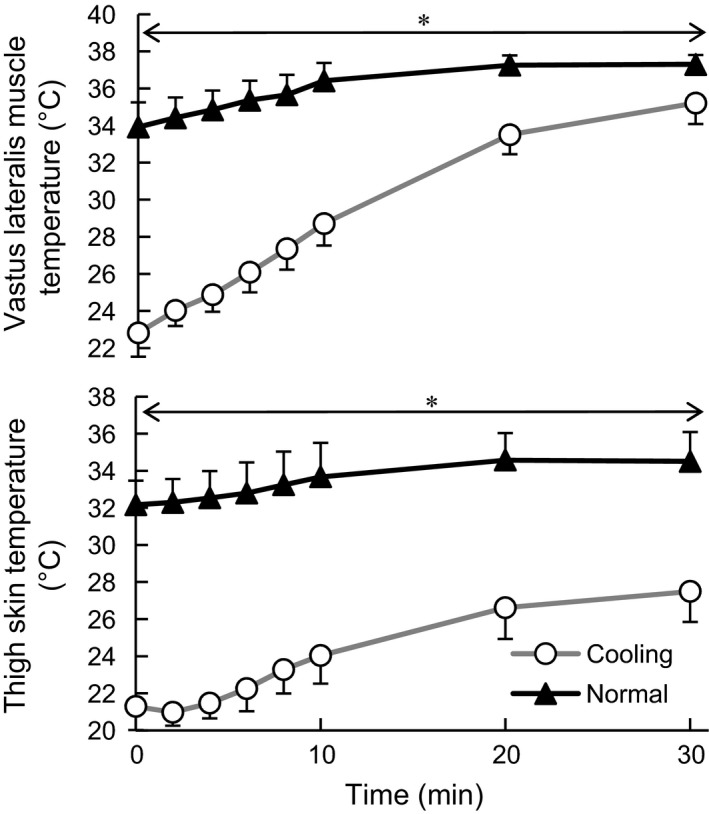
The vastus lateralis muscle temperature and thigh skin temperature during exercise. Values are mean ± SD of the cooling and normal conditions *Significant difference between conditions (*P* < 0.05).

*T*_muscle_ and *T*_thigh_ during exercise are shown in Figure 1. For both temperature variables, significant main effect of time (*F*_2.4, 16.6_ = 228.4, *η*_*p*_^2^ = 0.98, *P* < 0.001; *F*_1.7, 12.1_ = 69.0, $\eta_{p}^{2}$ = 0.99, *P* < 0.001), condition (*F*_1, 7_ = 623.8, $\eta_{p}^{2}$ = 0.99, *P* < 0.001; *F*_1, 7_ = 368.3, $\eta_{p}^{2}$ = 0.98, *P* < 0.001), and a significant interaction between those factors (*F*_2.1, 14.7_ = 75.1, $\eta_{p}^{2}$ = 0.99, *P* < 0.001; *F*_2.1, 14.9_ = 15.2, $\eta_{p}^{2}$ = 0.69, *P* < 0.001) were detected in the two‐way ANOVA. The lower body cold immersion decreased *T*_muscle_ to 22.7°C on average. After the onset of exercise *T*_muscle_ in C gradually recovered, though it was significantly lower than N throughout the 30‐min exercise (*P* < 0.05). *T*_thigh_ was about 21°C in C at the onset of exercise, then gradually increased during exercise, although it was significantly lower than that in N throughout the 30‐min exercise (*P* < 0.05).

### Pulmonary oxygen uptake and blood lactate concentration

**Figure 2:**
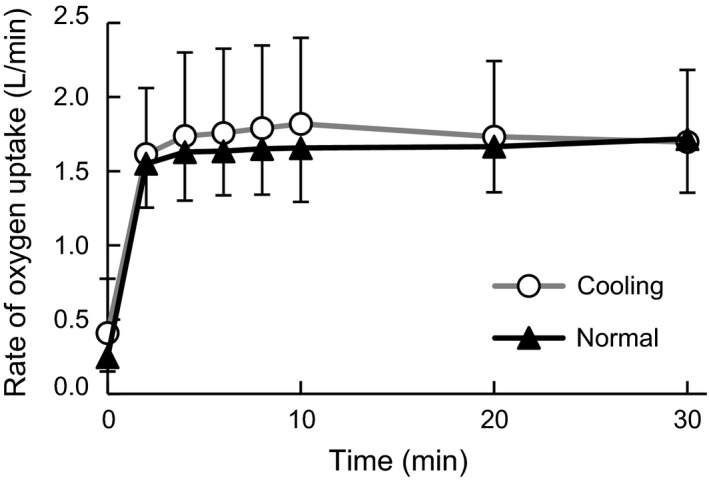
Change in the rate of oxygen uptake during the experiment. Values are mean ± SD of the cooling and normal conditions.

$\dot{V}$O_2_ during exercise showed a significant main effect of time (*F*_1.1, 8.0_ = 64.8, $\eta_{p}^{2}$ = 0.90, *P* < 0.001, Fig. 2). No significant difference was observed between conditions in $\dot{V}$O_2_ at any time point during exercise.

**Figure 3:**
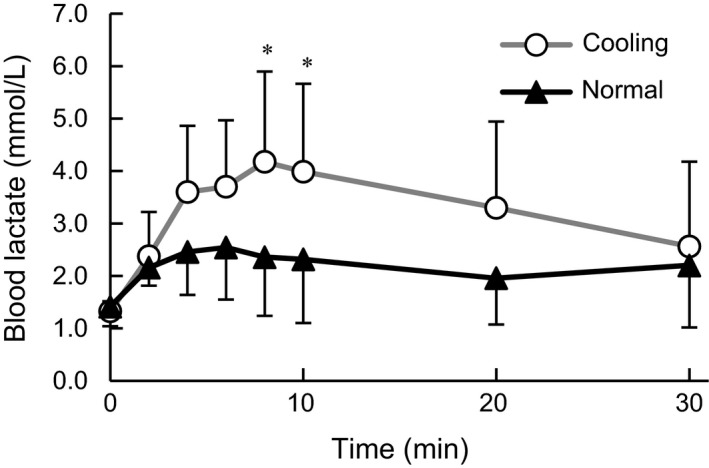
Change in the blood lactate concentration during exercise. Values are mean ± SD of each cooling and normal condition. * Significant difference between conditions (*P* < 0.05).

La showed significant main effect of time (*F*_1.9, 13.4_ = 10.1, $\eta_{p}^{2}$ = 0.59, *P* < 0.001), condition (*F*_1, 7_ = 20.3, $\eta_{p}^{2}$ = 0.74, *P* < 0.001), and a significant interaction between those factors (*F*_2.96, 20.7_ = 6.7, $\eta_{p}^{2}$ = 0.49, *P* < 0.001, Fig. 3). La was significantly higher in C than that in N at 8 min (*d *=* *1.25, *P* < 0.05) and 10 min (*d *=* *1.14, *P* < 0.05) after onset of exercise.

### Skeletal muscle oxygenation

**Figure 4:**
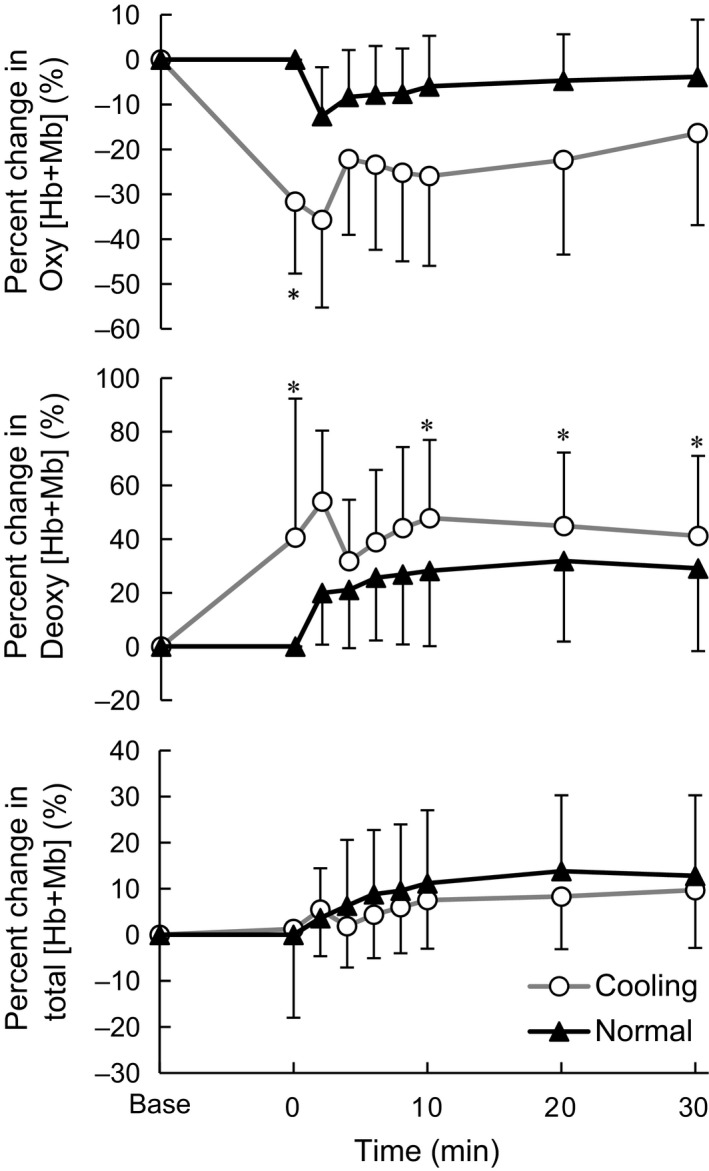
Percent change in the oxy, deoxy, and total hemoglobin and myoglobin in the vastus lateralis. Values are mean ± SD of each cooling and normal condition. *Significant difference between conditions (*P* < 0.05).

The percent change in Oxy, Deoxy, and total [Hb+Mb] in the vastus lateralis relative to the resting baseline before cooling are shown in Figure 4. The Oxy[Hb+Mb] showed significant main effect of time (*F*_1.8, 12.4_ = 5.7, $\eta_{p}^{2}$ = 0.45, *P* < 0.05) and condition (*F*_1, 7_ = 8.1, $\eta_{p}^{2}$ = 0.54, *P* < 0.05). At the onset of exercise the Oxy[Hb+Mb] in C was significantly lower than that in N (*d *=* *2.79, *P* < 0.01). The Deoxy[Hb+Mb] showed significant main effect of time (*F*_1.7, 11.7_ = 4.9, $\eta_{p}^{2}$ = 0.41, *P* < 0.05) and condition (*F*_1, 7_ = 17.9, $\eta_{p}^{2}$ = 0.72, *P* < 0.01). The Deoxy[Hb+Mb] was significantly higher in C at the onset of exercise (*d *=* *1.11, *P* < 0.05) and at 10 (*d *=* *0.69, *P* < 0.05), 20 (*d *=* *0.45, *P* < 0.05) and 30 min (*d *=* *0.40, *P* < 0.05) after the onset. The total [Hb+Mb] showed main effect of time (*F*_1.6, 11.5_ = 16.3, $\eta_{p}^{2}$ = 0.70, *P* < 0.001) and no difference was observed between conditions.

**Figure 5:**
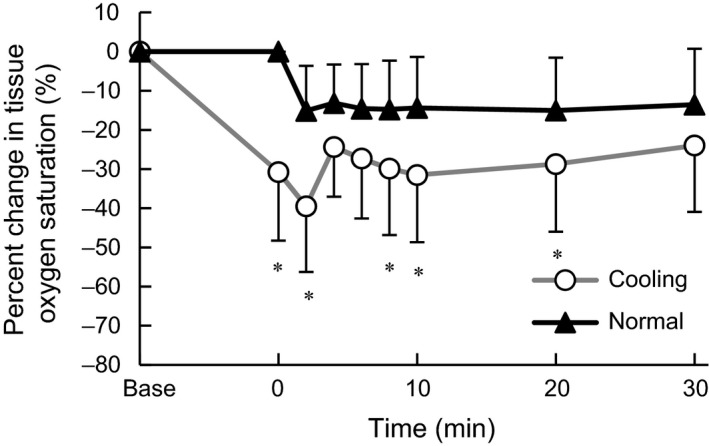
Percent change in the tissue oxygen saturation in the vastus lateralis. Values are mean ± SD of each cooling and normal condition. *Significant difference between conditions (*P* < 0.05).

The percent change in StO_2_ in the vastus lateralis relative to the resting baseline before cooling are shown in Figure 5. StO_2_ showed a significant main effect of condition (*F*_1.7, 11.7_ = 23.1, $\eta_{p}^{2}$ = 0.77, *P* < 0.001) and a significant interaction between those factors (*F*_2.0, 14.3_ = 5.2, $\eta_{p}^{2}$ = 0.43, *P* < 0.05). The percent change in StO_2_ was significantly lower in C at the onset of exercise (*d *=* *2.49, *P* < 0.05), 2 (*d *=* *1.70, *P* < 0.05), 8 (*d *=* *1.02, *P* < 0.05), 10 (*d *=* *1.12, *P* < 0.05), and 20 min (*d *=* *0.88, *P* < 0.05) after the onset.

### Kinetics of pulmonary oxygen uptake and muscle deoxy hemoglobin

**Figure 6:**
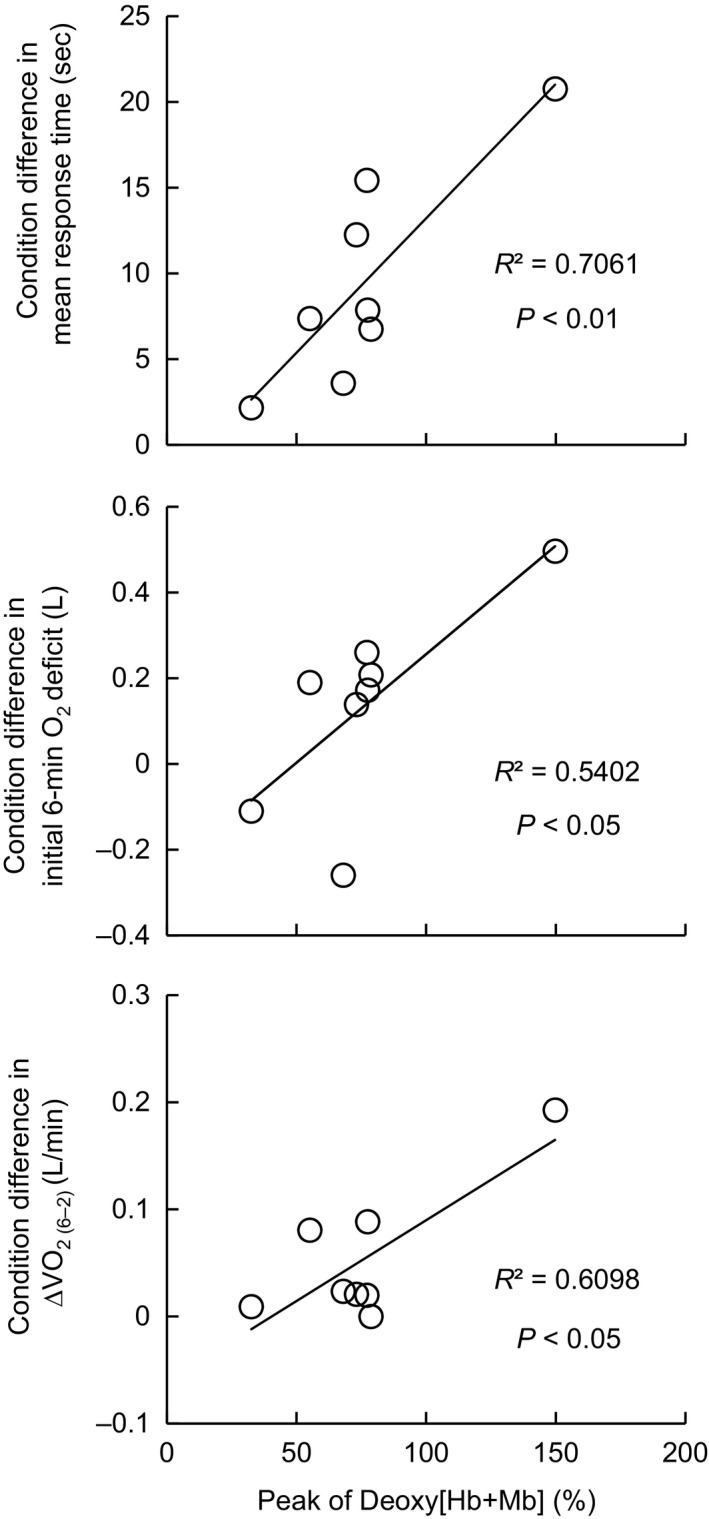
The condition differences in $\dot{V}$O_2_ mean response time, initial 6‐min O_2_ deficit, and ∆$\dot{V}$O_2 (6–2)_ as a function of the transient peak Deoxy[Hb+Mb] in the vastus lateralis in cooling condition. ∆$\dot{V}$O_2__(6–2)_: the increment in oxygen uptake between minutes 2 and 6.

The parameters of $\dot{V}$O_2_ kinetics during the first 6 min of exercise are shown in Table 1. Compared to N, condition C showed significantly longer time constant of $\dot{V}$O_2_ kinetics (*τ* $\dot{V}$O_2_) (*d *=* *1.23, *P* < 0.05) and MRT (*d *=* *1.23, *P* < 0.01). There were no significant differences between conditions in $\dot{V}$O_2 (base)_, Amp, TD, initial 6‐min O_2_ deficit or ∆$\dot{V}$O_2 (30–base)_. No difference was observed between Amp (i.e., ∆$\dot{V}$O_2_ between baseline and 6 min) and ∆$\dot{V}$O_2 (30–base)_ (i.e., Amp at the 30th min) in each condition. ∆$\dot{V}$O_2 (6–2)_ was significantly greater in C (*d *=* *0.97, *P* < 0.05) and significantly correlated with the La concentration averaged during 2 to 6 min (*r* = 0.71, *P* < 0.05). A transient peak in the Deoxy[Hb+Mb] (76.5 ± 33.5%) in the vastus lateralis was observed only in C at 41.3 ± 8.4 sec after onset of exercise. The peak Deoxy[Hb+Mb] in C was significantly correlated with differences between conditions in MRT, initial 6‐min O_2_ deficit and ∆$\dot{V}$O_2 (6–2)_ (*r* = 0.84, 0.74 and 0.80, respectively, *P* < 0.05, Fig. 6) and tended to be correlated with the *τ* $\dot{V}$O_2_ (*r* = 0.68, *P* = 0.06). The percentage of the *τ* $\dot{V}$O_2_ in C relative to N was significantly correlated with the peak Deoxy[Hb+Mb] in C (*r* = 0.71, *P* < 0.05).

**Table 1:** Oxygen uptake kinetics during the first 6 min of exercise

	Cooling	Normal
Baseline (L/min)	0.41 ± 0.37 [0.15–0.66]	0.25 ± 0.10 [0.18–0.31]
Amplitude (L/min)	1.28 ± 0.22 [1.13–1.48]	1.39 ± 0.30 [1.18–1.60]
Time delay (sec)	11.4 ± 10.7 [4.0–18.8]	16.3 ± 3.2 [14.1–18.5]
Time constant (sec)	39.0 ± 13.7 [29.5–48.5]	25.7 ± 6.8 [21.0–30.5]^a^
*R* ^2^ value of regression curve	0.84 ± 0.05 [0.81–0.88]	0.83 ± 0.10 [0.76–0.90]
Mean response time (sec)	45.6 ± 7.8 [40.2–51.0]	36.1 ± 7.7 [30.7–41.4]^a^
Initial 6‐min O_2_ deficit (L)	0.98 ± 0.30 [0.77–1.19]	0.85 ± 0.28 [0.66–1.04]
∆$\dot{V}$O_2__(6–2)_ (L/min)	0.09 ± 0.07 [0.04–0.14]	0.03 ± 0.03 [0.01–0.05]^a^
∆$\dot{V}$O_2__(30–base)_ (L/min)	1.29 ± 0.30 [1.08–1.50]	1.47 ± 0.35 [1.23–1.72]

Values are means ± standard deviation and [95% confidential interval].

a Significant difference between conditions (*P* < 0.05).

∆$\dot{V}$O_2__(6–2)_ and ∆$\dot{V}$O_2__(30–base)_: the increment in oxygen uptake between minutes 2 and 6, and between baseline and minute 30.

## Discussion

This study investigated the effect of muscle cooling on pulmonary $\dot{V}$O_2_ kinetics and muscle metabolism during exercise at LT work rate. The major finding of the present study was that at the onset of exercise, transient peak muscle deoxygenation was observed in C, which is associated with the delay of pulmonary $\dot{V}$O_2_ kinetics relative to N. Additionally, significantly higher La concentration was observed in C compared to N during exercise.

### Anaerobic metabolism in hypothermic skeletal muscle

After 30‐min cold immersion, the vastus lateralis *T*_muscle_ declined to 22.7°C on average, which was below *T*_muscle_ inducing impairment of muscle endurance and maximal force (Clarke et al. 1958; Heus et al. 1995; Wakabayashi et al. 2015). Since an elevation of La concentration was associated with lowering of *T*_muscle_ (Figs. 1 and 3), we suggest that the reduction of *T*_muscle_ would increase recruitment of glycolytic metabolism during exercise. Similar to current observation, greater anaerobic metabolism in cold water was reported in a previous study which showed higher minute ventilation to $\dot{V}$O_2_ ratio (V_E_/$\dot{V}$O_2_) during an incremental exercise test in cold (18°C) compared to warm water (Fujimoto et al. 2016). Since we set the LT work rate predetermined in normal condition for both conditions, participants actually exercised at LT work rate (moderate intensity) in N and above LT (heavy intensity) in C. The potential mechanism for the greater contribution of the glycolytic metabolism with hypothermic skeletal muscle is discussed as follows.

In this study no direct measurement of muscle blood flow was conducted; however, cold‐induced muscle vasoconstriction (Rennie et al. 1980) seemed to be one of the major factors inducing the anaerobic metabolism. However, the percent change in the total [Hb+Mb] indicates no difference between conditions in the microvascular Hb volume and muscle O_2_ diffusion capacity (Fukuoka et al. 2015). The significantly lower StO_2_ in C might be attributed in part to less blood perfusion in the hypothermic skeletal muscle (Rennie et al. 1980; Thorsson et al. 1985; Gregson et al. 2011) and leftward shift of oxyhemoglobin dissociation curve with lower temperature (Severinghaus 1958; Astrup et al. 1965). The larger increase of Deoxy[Hb+Mb] in C during exercise suggests the lower muscle blood flow/O_2_ delivery relative to O_2_ consumption (Koga et al. 2012, 2015). Based on these observations by the NIRS method, less tissue oxygenation (lower StO_2_) due to lower O_2_ delivery relative to O_2_ consumption (higher Deoxy[Hb+Mb]) might induce the greater anaerobic contribution in C. Additionally, lower blood perfusion may also impair the lactate utilization in the skeletal muscle (Gladden et al. 1992; Gladden 2000) resulting in the greater La concentration in C.

In addition to differences in O_2_ transport to the working muscle due to the cold‐induced muscle vasoconstriction, reduction of *T*_muscle_ *per se* slows ATP utilization (Edwards et al. 1972) and slows Ca^2+^ release and uptake from the sarcoplasmic reticulum (Kossler et al. 1987; Herve et al. 1992). Previous studies have also reported that a greater number of motor units were recruited in cold environment in order to maintain the same work load compared to neutral conditions (Mallette et al. 2018). Because of the greater cold sensitivity and lower power output of slow‐twitch fibers (Ranatunga 1984), fast‐twitch fibers were recruited to a greater extent in a cold environment compared to warm condition even at moderate‐intensity exercise in scup (Rome et al. 1992). Thus, the greater anaerobic contribution with hypothermic skeletal muscle might be attributed to the relatively more recruitment of the fast‐twitch fibers relying more on glycolytic energy transfer, which may be associated to the larger $\dot{V}$O_2_ slow component (see below).

### Kinetics of oxygen uptake and Deoxy[Hb+Mb] at the onset of exercise

At the onset of exercise (i.e., at the time of the lowest *T*_muscle_ was recorded), the longer *τ* $\dot{V}$O_2_ and MRT observed in C was consistent with a previous study (Shiojiri et al. 1997). Shiojiri et al. (1997) suggested that the delay of $\dot{V}$O_2_ kinetics was due to decreased O_2_ extraction and/or impairment of oxidative reactions based on similar cardiac output in C and N conditions. In the present study, the transient peak in Deoxy[Hb+Mb] in C indicates possible greater O_2_ extraction fraction (Koga et al. 2012, 2014) and less oxygen delivery to muscle oxygen uptake. The different interpretation for the O_2_ extraction between the present and previous studies (Shiojiri et al. 1997) might be attributed to exercise intensity, that was above anaerobic threshold (AT) in the present cooling condition, while below AT in the previous study (Shiojiri et al. 1997). The lower blood flow to the working muscle in cold (Rennie et al. 1980) might have been reflected in the transient peak of Deoxy[Hb+Mb] and the lower StO_2_ in C (Figs. 4 and 5). The peak Deoxy[Hb+Mb] was correlated with the ∆MRT between conditions and *τ* $\dot{V}$O_2_ in C relative to N. Additionally, the condition differences in O_2_ deficit and ∆$\dot{V}$O_2 (6–2)_ were also strongly correlated with the peak Deoxy[Hb+Mb] in C (Fig. 6). Based on these observations, we suggest that the slower $\dot{V}$O_2_ kinetics in C would be caused by the limitation of aerobic energy provision due to less blood perfusion in the working muscle. Similar to the present study, Bowen et al. (2013) reported delay of $\dot{V}$O_2_ kinetics in hypoxia and correlation with transient peak of skeletal muscle Deoxy[Hb+Mb] assessed using time resolved near‐infrared spectroscopy. In terms of lower O_2_ delivery to the working muscle, a similar mechanism in the slowed $\dot{V}$O_2_ kinetics is plausible in a hypoxic environment and muscle cooling condition. For example, there have been several studies reporting delay of $\dot{V}$O_2_ kinetics with interventions to limit O_2_ delivery to the working muscle (Hughson and Smyth 1983; Hughson et al. 1991; Goodwin et al. 2012).

As explained above, fast‐twitch fibers might be recruited more in the cooling condition (Ranatunga 1984; Rome et al. 1992). Relating to previous studies (Barstow et al. 1996; Pringle et al. 2003) reporting delay of $\dot{V}$O_2_ kinetics in participants with a greater percentage of fast‐twitch fibers, slower $\dot{V}$O_2_ kinetics observed in C of the present study might indicate the greater recruitment of fast‐twitch fibers in C. Additionally, although the exercise intensity was set at the same absolute work rate for both N and C, the larger ∆$\dot{V}$O_2 (6–2)_ in C was observed as shown (Table 1). Concerning the significant correlation between the ∆$\dot{V}$O_2 (6–2)_ and La concentration averaged during 2 to 6 min, the larger recruitment of glycolytic metabolism in C would increase slow component and delay $\dot{V}$O_2_ kinetics (Whipp and Wasserman 1986; Wasserman 1987).

### Methodological limitation

In this study, we choose the LT work rate predetermined in normal condition for assessing recruitment of anaerobic metabolism with hypothermic skeletal muscle even with a slight increase over the threshold in C. Thus, participants might exercise in moderate and heavy intensity domains in N and C, respectively. It might have been more appropriate to have participants exercising in the moderate‐intensity domain (e.g., at 80% LT) in both conditions to determine whether anaerobic contribution was elevated in C but not in N, and also whether $\dot{V}$O_2_ primary kinetics slowed and/or slow component was created in C during moderate‐intensity exercise.

## Conclusion

One of the major findings in this study was that exercise with hypothermic skeletal muscle increased recruitment of glycolytic metabolism as shown in the significantly higher blood lactate concentration even at lactate threshold workload that was predetermined in thermoneutral environment. Our second major finding was the delay of oxygen uptake kinetics at the onset of exercise after pre‐cooling and a significant correlation with the transient peak of deoxy hemoglobin in the working muscle.

## Conflict of Interest

None declared.
